# Recent advances in methods to assess the activity of the kinome

**DOI:** 10.12688/f1000research.10962.1

**Published:** 2017-06-26

**Authors:** Maria Radu, Jonathan Chernoff

**Affiliations:** 1 Cancer Biology Program, Fox Chase Cancer Center, 333 Cottman Avenue, Philadelphia, PA, 19111-2497, USA

**Keywords:** kinome, enzymes, cancer therapeutics, assays

## Abstract

Protein and lipid kinases are deregulated in most, if not all, cancers and are among the most valuable therapeutic targets in these diseases. Despite the introduction of dozens of effective kinase inhibitors into clinical practice, the development of drug resistance remains a major barrier to treatment because of adaption of cellular signaling pathways to bypass targeted kinases. So that the basal and adaptive responses of kinases in cancer can be better understood, new methods have emerged that allow simultaneous and unbiased measurement of the activation state of a substantial fraction of the entire kinome. Here, we discuss such kinome-profiling methodologies, emphasizing the relative strengths and weaknesses of each approach.

## Introduction

Cancer is a genetic disease caused by mutational damage to DNA or by aberrant epigenetic events, but the properties associated with malignant cells are mediated by subsequent changes in the activity and organization of signaling proteins that directly regulate cell proliferation, survival, secretion, and the ability to invade and metastasize. Among these signaling proteins, none plays a more central role in cancer than the protein and lipid kinases, which regulate a vast number of cellular processes. For this reason, determining kinase activities and identifying drugs that suppress or, more rarely, activate these enzymes have become the subjects of intense interest, and many small-molecule kinase modulators have made significant impact as cancer therapeutics ^1– 3^.

Although the significance of altered kinase signaling to carcinogenesis and to drug resistance is widely recognized, our ability to determine the signaling status of cells lags behind our ability to sequence, quantitate, and interpret changes in DNA and RNA. There are important limitations to our current methods to analyze kinase-driven signaling pathways because, unlike nucleic acid sequence analysis, the study of enzymes is not intrinsically digital, and there are no generalized, gold-standard methods to measure the activity of kinases *en masse*. As such, we not only lack knowledge of the baseline signaling state in tumors but also cannot conveniently measure how signaling pathways adapt and rewire as cells become resistant to a given targeted therapy. This inability in turn limits our ability to predict how tumors will respond to drugs designed to modulate kinase activity and how best to combine such agents in a given tumor to overcome resistance.

## Current methods used to study the kinome

The mammalian genome encodes more than 500 protein and lipid kinases (collectively termed the kinome), of which several hundred may be expressed in a given cell type ^4^. Two general approaches have emerged to assess the activity and architecture of the kinome in cells: one based on activation state-specific antibodies and another based on mass-spectroscopic analysis of phosphorylated substrates or of kinases captured in their activated state on inhibitor-coated beads ^5^. These methods each have particular strengths and weaknesses, and their use will be dictated by the particulars of experimental design, as described below.

## Antibody-based arrays

When activated, almost all protein kinases are phosphorylated at one or more key residues within the activation loop, resulting in rearrangement of the catalytic site into an active conformation ^6, 7^. For this reason, assays employing recognition by phospho-specific antibodies directed against these activation loop sites have been widely used as surrogates for kinase activity assays. In forward-phase protein arrays, well-characterized activation state-specific antibodies, usually clustered by signaling pathways, are spotted in a matrix on a solid support and incubated with cellular lysates, and bound proteins are detected either by a fluorescent secondary antibody or by a previously attached fluorescent tag. Reverse-phase protein arrays use a similar overall design, but in this case it is the cellular lysates that are immobilized on an array platform, which then are probed with specific phospho-antibodies ^8^. Such assays have been used to analyze kinase activity in a variety of cancers and offer a relatively low-cost and potentially high-throughput method to assess the kinome ^9^; however, these methods are limited by antibody specificity, sensitivity, and availability. In addition, the activity of some protein kinases cannot be assessed by phospho-antibodies, as they are constitutively phosphorylated in the activation loop (for example, PKA, PAK4, PKCζ, and PKCθ) ^10– 12^.

## Mass spectrometry-based assays

The use of mass spectrometry (MS)-based approaches to investigate the activity of the kinome is an increasingly viable alternative to using antibody-based methods. Here, we will restrict our comments to the use of MS to assess kinase activity directly, as opposed to its use to identify cellular phosphoproteins in general, as the latter dataset provides, at best, an indirect map to the activity of individual kinases.

## Kinase activity assay for kinome profiling

**Figure 1.**
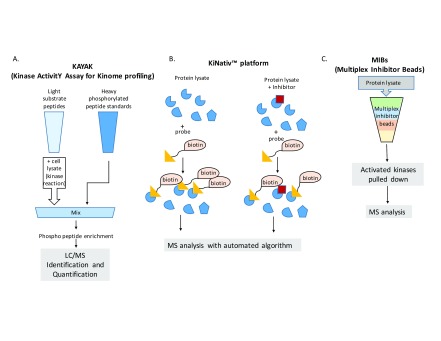
Mass spectrometry-based assays for kinome profiling. ( **A**) Kinase activity assay for kinome profiling (KAYAK) methodology compares phosphorylation of a defined peptide substrate library upon incubation with cell lysates with a set of identical phosphorylated “heavy” standards. ( **B**) KiNativ™ platform enriches kinases present in a cell lysate by capturing the conserved lysine of kinases on beads harboring an acyl phosphate group bound to biotin. ( **C**) Multiplexed inhibitor beads (MIBs) are multilayered kinase inhibitor beads that are able to bind active kinases while enriching for the low-expressed kinases present in a cell lysate mixture. LC, liquid chromatography; MS, mass spectrometry.

Kinase activity assay for kinome profiling (KAYAK) methodology relies on the known substrate preferences of various protein kinases that are dictated by motifs surrounding the site of phosphorylation. In the most recent incarnation of this method, libraries of peptides representing many well-characterized substrate motifs for kinases of interest are incubated with a cell lysate in the presence of ATP, then mixed with matching phosphorylated, SILAC (stable isotope labeling with amino acids in cell culture)-labeled “heavy” peptides, and analyzed by liquid chromatography/MS. The activity of kinases in the lysate is inferred from the peptides that are phosphorylated. The advantages of this assay are its ease of deployment and its semi-quantitative nature. These strengths are counterbalanced by its relatively low sensitivity and a reliance on the assumption that a given peptide accurately reports on the activity of its matching kinase. As the substrate preferences for many kinases are not known, this assumption may not prove valid in certain cases, so the results of this method need to be interpreted with appropriate caution ^13, 14^ ( Figure 1A).

## KiNativ™ platform

Various kinase capture methods have been used in tandem with MS to assess kinase activity. For example, the KiNativ™ platform uses specific ActivX beads to pull down kinases. This method takes advantage of the conserved lysine residue that is present in the ATP-binding site of all kinases. The KiNativ™ method uses a probe that has an acyl phosphate group bound to a biotin tag. The lysine in the ATP pocket of the bound kinase forms a covalent bond with the acyl group. Owing to the apposition of active biotin to nearby lysine residues in the catalytic cleft, the kinase is labeled by the biotin tag and subsequently can be identified by MS ^15^ ( Figure 1B). In a further improvement of the assay, Adachi *et al*. used an ATP and ATP probe-competitive approach that increased the specificity of kinases bound to ActivX beads ^16^.

One drawback of the ActivX format, an otherwise useful method, is that it binds equally to active and inactive protein kinases and thus cannot be used alone to assess the activity status of these enzymes. Also, quantitative MS can be difficult to achieve because of variations in peak intensity or peak area that also vary with primary protein structure or sample complexity. To overcome this hurdle, an automated algorithm has been developed and employed to aid in quantitating MS peaks obtained from tagged kinases in the presence and absence of inhibitors ^17, 18^. Even with this advance, the main utility of ActivX is for kinase enrichment rather than for measuring kinase activity.

## Multiplexed kinase inhibitor beads

A different approach to kinome profiling involves the use of beads linked to kinase inhibitors, sometimes termed “kinobeads” ^19^ or “multiplexed inhibitor beads” (MIBs) ^5, 20– 29^. MIBs are arranged in layers on the basis of kinase specificity: specific kinase inhibitors in the first layer capture the more abundant kinases, whereas the less abundant kinases are captured in subsequent layers by pan-kinase inhibitors. For both kinobeads and MIBs, the immobilized type I ATP-like protein kinase inhibitors act as traps for activated kinases present in the protein lysate under study. Although such beads capture ATP-binding proteins in general, subsequent MS analysis can determine which peptides derive from protein kinases. The key advantage of this method is that, for the majority of kinases, the immobilized inhibitors bind these enzymes only when they are in the “DFG-in” active conformation ^20, 21^. However, it should be remembered that several factors affect kinase capture, including overall kinase expression levels and the affinity of the immobilized probes ^30^. For these reasons, it is essential to confirm the identified activated kinases by secondary methods. Despite these caveats, MIB technology has proven to be a useful approach, as it bypasses the limitations of antibody-based arrays and it requires no *a priori* knowledge of candidate kinases and no specialized reagents other than the inhibitor beads, which are uniform and can be mass produced. Another important advantage of this method is that one can measure endogenous kinase activity in minimally processed lysates. To date, optimized versions of this procedure have been used to capture more than half of all human kinases in a single assay ^27, 31^.

The MIB assay has been used successfully to study the adaptive response of the kinome following drug exposure ^27, 28^. The versatility, high throughput, and ability to monitor the dynamic nature of the kinome in response to drug treatment or any other type of external stimuli make the use of MIBs a valuable option in screening for drug resistance events. MIB kinase enrichment has also been used successfully with an improved chromatography detection using C18 silica columns to provide insights into modulations in signaling pathway activity following exposure to various inhibitors ^32^.

Attempts to introduce a more accurate quantitation to the MIB procedure include the use of SILAC-labeled kinase standards and isobaric tags such as isobaric tags for relative and absolute quantitation (iTRAQ) labeling ^20, 21, 33, 34^. These methods are currently the most common quantitative MS approaches used for proteomic and kinomic studies in combination with chemical proteomic assays ^35^.

## A look forward

To date, most attempts to target anti-neoplastic agents to individual cancers have relied on genomic or transcriptomic analyses. However, recent advances in proteomic technologies suggest that a more complete picture of tumors is feasible and potentially useful in cancer diagnosis and therapy. Already, combining genomic with quantitative proteomic analyses has proven to be a powerful approach in triple-negative breast cancers, ovarian cancers, and other malignancies. In some cases, such “proteogenomic” approaches have identified tumor vulnerabilities that could not have been predicted on the basis of genomic analysis alone ^36– 39^. It is to be hoped that the addition of kinome profiling to existing nucleic acid-based methods will help address important, unanswered questions in oncology, such as why some patients become resistant to therapy whereas others do not, why oncogenic mutations drive different pathways in different cancers, and what groups best benefit from which targeted therapies.

As sensitivity improves, it is also likely that kinome and phospho-proteome profiling will be available with minute amounts of biological material, such that serial screening of a tumor’s proteome can detect reprograming events and predict drug sensitivity in small samples such as needle aspirates or circulating tumor cells. Single-cell proteomics have advanced significantly ^40^ and are yet another possible alternative to better understanding the activation-inactivation balance in signaling pathways in small sample sizes. However, whatever technical improvements are made, there will remain a need for a more in-depth understanding of the biochemical and structural characteristics of kinases that drive cancers and other disorders, such as their modes of regulation and substrate specificity.

Finally, we believe that a combination of advanced proteomic and functional assays in tandem with computational models will serve as the new platform to better identify and target driver kinases, and the signaling networks they support, in cancer and other diseases. This will allow additional and increasingly more specific therapies that are directed at the activated networks or signaling hubs that sustain pathologic activity.
